# The Impact of Multiplex PCR in Diagnosing and Managing Bacterial Infections in COVID-19 Patients Self-Medicated with Antibiotics

**DOI:** 10.3390/antibiotics11040437

**Published:** 2022-03-24

**Authors:** Iulia Bogdan, Cosmin Citu, Felix Bratosin, Daniel Malita, Ioan Romosan, Camelia Vidita Gurban, Adrian Vasile Bota, Mirela Turaiche, Melania Lavinia Bratu, Ciprian Nicolae Pilut, Iosif Marincu

**Affiliations:** 1 Methodological and Infectious Diseases Research Center, Department of Infectious Diseases, “Victor Babes” University of Medicine and Pharmacy, Eftimie Murgu Square 2, 300041 Timisoara, Romania; iulia.georgianabogdan@gmail.com (I.B.); felix.bratosin7@gmail.com (F.B.); gurban.camelia@umft.ro (C.V.G.); bota.adrian1@yahoo.com (A.V.B.); mirela.turaiche@gmail.com (M.T.); bratu.lavinia@umft.ro (M.L.B.); pilut.ciprian@umft.ro (C.N.P.); imarincu@umft.ro (I.M.); 2 Department of Radiology, “Victor Babes” University of Medicine and Pharmacy, Eftimie Murgu Square 2, 300041 Timisoara, Romania; malita.daniel@umft.ro; 3 Department of Internal Medicine, “Victor Babes” University of Medicine and Pharmacy, Eftimie Murgu Square 2, 300041 Timisoara, Romania; romosan.ioan@umft.ro; 4 Department of Biochemistry, “Victor Babes” University of Medicine and Pharmacy, Eftimie Murgu Square 2, 300041 Timisoara, Romania; 5 Department of Psychology, “Victor Babes” University of Medicine and Pharmacy, Eftimie Murgu Square 2, 300041 Timisoara, Romania; 6 Multidisciplinary Research Center on Antimicrobial Resistance (MULTI-REZ), Microbiology Department, “Victor Babes” University of Medicine and Pharmacy, Eftimie Murgu Square 2, 300041 Timisoara, Romania

**Keywords:** COVID-19, SARS-CoV-2 infection, multiplex PCR, self-medication, antibiotics

## Abstract

The multiplex PCR is a powerful and efficient tool that was widely used during the COVID-19 pandemic to diagnose SARS-CoV-2 infections and that has applications for bacterial identification, as well as determining bacterial resistance to antibiotics. Therefore, this study aimed to determine the usability of multiplex PCR, especially in patients self-medicated with antibiotics, where bacterial cultures often give false-negative results. A cross-sectional study was developed in two COVID-19 units, where 489 eligible patients were included as antibiotic takers and non-antibiotic takers. Antibiotic takers used mostly over-the-counter medication; they suffered significantly more chronic respiratory conditions and were self-medicated most often with cephalosporins (41.4%), macrolide (23.2%), and penicillin (19.7%). The disease severity in these patients was significantly higher than in non-antibiotic takers, and bacterial superinfections were the most common finding in the same group (63.6%). Antibiotic takers had longer hospital and ICU admissions, although the mortality rate was not significantly higher than in non-antibiotic takers. The most common bacteria involved in secondary infections were *Staphylococcus aureus* (22.2%)*, Pseudomonas aeruginosa* (27.8%), and *Klebsiella* *spp* (25.0%). Patients self-medicating with antibiotics had significantly higher rates of multidrug resistance. The multiplex PCR test was more accurate in identifying multidrug resistance and resulted in a quicker initiation of therapeutic antibiotics compared with instances where a bacterial culture was initially performed, with an average of 26.8 h vs. 40.4 h, respectively. The hospital stay was also significantly shorter by an average of 2.5 days when PCR was used as an initial assessment tool for secondary bacterial infections. When adjusted for age, COVID-19 severity, and pulmonary disease, over-the-counter use of antibiotics represented a significant independent risk factor for a prolonged hospitalization (AOR = 1.21). Similar findings were observed for smoking status (AOR = 1.44), bacterial superinfection (AOR = 1.52), performing only a conventional bacterial culture (AOR = 1.17), and a duration of more than 48 h for bacterial sampling from the time of hospital admission (AOR = 1.36). Multiplex PCR may be a very effective method for diagnosing secondary bacterial infections in COVID-19 individuals self-medicating with antibiotics. Utilizing this strategy as an initial screen in COVID-19 patients who exhibit signs of sepsis and clinical deterioration will result in a faster recovery time and a shorter period of hospitalization.

## 1. Introduction

Coronavirus disease 2019 (COVID-19), caused by the severe acute respiratory syndrome coronavirus-2 (SARS-CoV-2), was initially identified in late 2019 and quickly developed into a pandemic, as stated on 11 March 2020 by the World Health Organization [1]. While the majority of COVID-19 cases present with moderate or simple respiratory diseases, patients may develop complications such as coinfections, acute respiratory distress syndrome, or sepsis [2,3]. The yet unknown effect size of coinfection rates between SARS-CoV-2 and other respiratory diseases, along with the fast worldwide spread of the virus and its variations, necessitates the establishment of a long-term diagnostic technique that is both efficient and sustainable [4]. Coinfections are common in patients with viral respiratory diseases such as influenza, and current recommendations advocate empiric antibiotic therapy and coinfection testing in patients with a severe clinical history of influenza infection [5], although coinfections and superinfections in patients with influenza seem to occur at higher rates than in those with SARS-CoV-2 [6]. Nevertheless, coinfection rates may be greater than predicted in COVID-19, and variations can occur depending on the variant involved, posing a significant diagnostic and therapeutic challenge for doctors. Numerous investigations have shown a broad range of coinfection frequencies in SARS-CoV-2 patients, ranging from 3% to more than 20% depending on the population investigated [7,8]; however, precise information on community-acquired bacterial coinfections is insufficient.

Coinfections occur simultaneously with the initial SARS-CoV-2 infection, while superinfections develop during the disease’s clinical course. However, these two entities are often not described explicitly in the literature, resulting in inconsistent reporting of the rates of coinfections and superinfections in COVID-19 patients [9]. According to one meta-analysis, 3.5% of COVID-19 patients had a bacterial coinfection before admission, and 14.3% acquired a bacterial superinfection during their hospital stay, while more than 70% of all patients got empirical antibiotic therapy [10]. COVID-19 patients treated in the intensive care unit (ICU) had a greater prevalence of bacterial superinfections than patients treated in conventional wards, according to observational data. Intriguingly, ventilator-associated pneumonia rates are much higher in COVID-19 patients, ranging from 16% to 78% [11], compared to non-COVID-19 patients, where the incidence is roughly 10%, depending on the underlying population [12,13]. Coinfections are most often caused by *Streptococcus pneumoniae, Staphylococcus aureus, Mycoplasma pneumoniae*, influenza A, parainfluenza, rhinovirus, enterovirus, respiratory syncytial virus (RSV), and other coronaviruses [14,15]. Current data show that coinfections with other respiratory viruses may exacerbate the illness course, resulting in increased disease severity and death. Thus, it is critical to identify the pathogens in coinfected COVID-19 individuals and assess their influence on clinical outcomes.

Multiplex polymerase chain reaction (PCR) panels may promptly detect the presence of respiratory infections and may aid in defining antimicrobial indications and medication selection [16]. Whenever possible, sampling of upper and lower respiratory tract secretions, such as nasopharyngeal or oropharyngeal exudate, bronchoalveolar lavage fluid, or endotracheal aspirate samples, is critical for detecting organisms and determining their bacterial load in COVID-19 patients who develop complications such as hospital-acquired pneumonia or ventilator-associated pneumonia [17,18]. In this context, recent multiplex PCR-based or array-based multi-pathogen detection tests, as well as, in certain cases, antibiotic resistance gene detection assays, aid in the diagnosis of bacterial infections subsequent to or concurrent with SARS-CoV-2 infection in patients with COVID-19 [19].

Antibiotic resistance is a serious danger since every year resistant bacteria claim at least 700,000 lives worldwide [20]. Antibiotic self-medication is a significant contributor to antimicrobial resistance, and it is characterized as an illogical and improper use of antibiotics by people who self-diagnose their symptoms/illnesses and treat them without prescriptions, medical advice, or supervision [21]. Prior studies on the variables influencing antibiotic self-medication have concentrated on an individual’s knowledge of antibiotics, antibiotic-use behaviors, accessibility to antibiotics, and demographic features [22]. While COVID-19 has been shown to substantially affect people’s mental health, psychological discomfort linked with perceived health concerns is likely one of the primary reasons for antibiotic self-medication during the COVID-19 pandemic. As such, the purpose of this retrospective multicentric observational study was to determine whether routine multiplex PCR-based screening for secondary-acquired bacterial pathogens could assist physicians in identifying secondary bacterial infections with greater accuracy in patients self-medicating with antibiotics and in predicting the clinical course of COVID-19.

## 2. Results

### 2.1. General Characteristics

Data collection identified 489 eligible patients that were hospitalized for COVID-19 and suffered a secondary bacterial infection. These included 198 individuals that admitted self-medicating with antibiotics since the onset of symptoms. The comparison of the background data from the group of antibiotic takers and non-antibiotic takers in Table 1 did not identify many significant differences, except for the personal history of pulmonary diseases, which were more prevalent in the group of antibiotic takers. A total of 47 (23.7%) patients taking antibiotics suffered from chronic bronchitis, compared with 39 (13.4%) in the group of non-antibiotic takers (*p*-value = 0.003). Similarly, there were 24 (12.1%) patients taking antibiotics who suffered from chronic obstructive pulmonary disease (COPD), compared with 17 (5.8%) in the group of non-antibiotic takers (*p*-value = 0.013).

**Figure 1:**
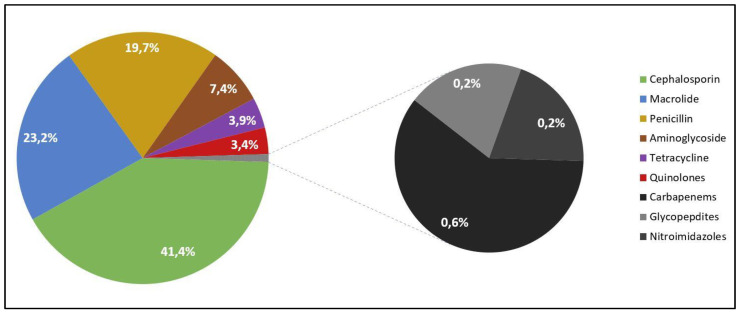
Frequency of self-medicated antibiotics among patients with COVID-19 and secondary bacterial infections.

Of the 198 patients with COVID-19 and a secondary bacterial infection, the majority self-medicated with cephalosporins (41.4%), followed by macrolide (23.2%), and penicillin (19.7%), as presented in Figure 1. The least common antibiotics taken at home were carbapenems (0.6%), glycopeptides (0.2%), and nitroimidazoles (0.2%). The source of this medication was mostly over-the-counter (67.2%).

### 2.2. Outcomes

An in-depth analysis of COVID-19 patient outcomes determined multiple statistically significant differences between patients who self-medicated with antibiotics and non-antibiotic takers (Table 2). There were significantly more cases of ventilator-associated pneumonia in the group of antibiotic takers (6.1% vs. 2.4%, *p*-value = 0.040). The proportions of secondary bacterial infections were statistically significantly different, where bacterial superinfections represented the highest proportion in antibiotic takers (63.6%), compared with 52.6% in non-antibiotic takers (*p*-value = 0.015). The COVID-19 severity and lung involvement were higher in the group of antibiotic takers, where ground glass opacities spread over more than 60% of the pulmonary area were seen in 11.1%, compared with 6.2% in non-antibiotic takers (*p*-value = 0.022). Additionally, 24 (12.1%) of the antibiotic takers developed a severe SARS-CoV-2 infection, compared with 21 (7.2%) in the non-antibiotic takers group (*p*-value = 0.047). In the group with the highest number of severe SARS-CoV-2 infections there was a greater need for invasive ventilation (10.6% vs. 5.6%, *p*-value = 0.023), and ICU admissions (9.6% vs. 4.8%, *p*-value = 0.038). The same patients who self-medicated with antibiotics had a longer stay in the ICU (12.9 vs. 11.6, *p*-value = 0.014) and a higher duration between symptom onset and death (15.2 days vs. 13.7 days, *p*-value = 0.009). However, overall mortality did not differ significantly between the two main study groups.

The comparison of the biological parameters of the two independent study groups presented in Table 3, identified significantly higher values for kidney and liver function tests in antibiotic takers, indicating organ dysfunction. A statistically significant higher average value of creatinine and blood urea nitrogen was observed in the group of antibiotic takers (1.36 vs. 1.28, *p*-value = 0.004), respectively (9.1 vs. 8.6, *p*-value = 0.048). ALT and AST levels were also significantly increased in patients that self-medicated with antibiotics (53 vs. 49, *p*-value = 0.012), respectively (44 vs. 42, *p*-value = 0.016). Other significant differences in the biological parameters were determined in the study groups’ median values of the inflammatory markers IL-6 (42 vs. 37, *p*-value = 0.016), and fibrinogen (5.1 vs. 4.5, *p*-value = 0.003).

**Figure 2:**
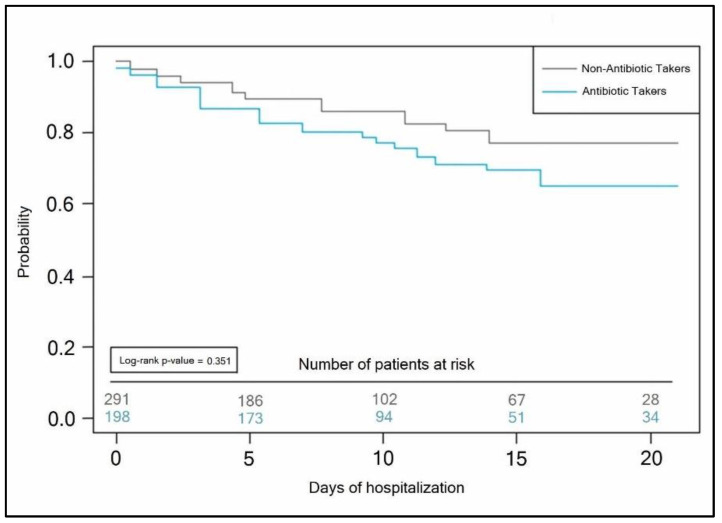
Kaplan–Meyer probability curve of hospitalization duration by the antibiotic use status of COVID-19 patients.

A Kaplan–Meyer curve was plotted in Figure 2 to determine the probability of the hospitalization duration in patients with COVID-19 stratified by antibiotic use. There was no significant difference between antibiotic-takers and non-antibiotic takers in the number of days until discharge (Log-rank *p*-value = 0.351).

### 2.3. Microbial Identification

The parallel bacterial identification by multiplex RT-PCR and conventional culture techniques was performed in both antibiotic takers and non-antibiotic takers. PCR testing was significantly more accurate in identifying bacteria in the group of antibiotic takers. There were 13.7% false-negative results in the group of antibiotic takers tested by PCR, compared with 48.0% false-negative results when tested by bacterial culture (*p*-value < 0.001). On the contrary, the PCR and bacterial culture methods did not show significant differences in false-negative results in the group of non-antibiotic takers (12.1% vs. 17.1%, *p*-value = 0.104). Sputum samples were positive in 91.7% of specimens analyzed with PCR, and only 51.4% when analyzed via conventional cultures (*p*-value < 0.001). Similarly, the PCR test was significantly more accurate in blood and urine samples taken from the same 72 antibiotic takers with COVID-19 (Table 4). There was no significant difference in the group of non-antibiotic takers regarding the accuracy of identifying bacteria from different types of samples.

The most commonly involved pathogen in the group of antibiotic takers was *Klebsiella spp.* which was identified in 25.0% of cases by PCR and 12.5% of cases by bacterial culture in the same group of patients. Statistically significant differences were observed in the accuracy of the tests involved when identifying *Staphylococcus aureus* (22.2% by PCR vs. 9.7% by culture, *p*-value = 0.040), and *Pseudomonas aeruginosa* (27.8% by PCR vs. 12.5% by culture, *p*-value = 0.022). In the group of non-antibiotic takers, the only significant difference observed was the proportion of positive tests for *Enterococcus faecalis* (20.2% by PCR vs. 9.6% by culture, *p*-value = 0.040).

The multiplex RT-PCR proved its efficiency in determining antibiotic resistance, where significant changes were only observed in the group of antibiotic takers (Table 4). A total of 43.1% of the samples tested by PCR were resistant to cephalosporins, compared with 26.4% identified by culture in the same patients (*p*-value = 0.035). Other significant differences were in macrolide resistance (38.9% tested by PCR vs. 16.7% tested by culture, *p*-value = 0.002). Additionally, multidrug resistance was more prevalent in the group of 198 antibiotic takers hospitalized with COVID-19 (91.7% by PCR vs. 79.2% by culture, *p*-value = 0.033). Lastly, the same group was found with a significantly higher number of infections with more than two pathogens (*p*-value = 0.049).

**Figure 3:**
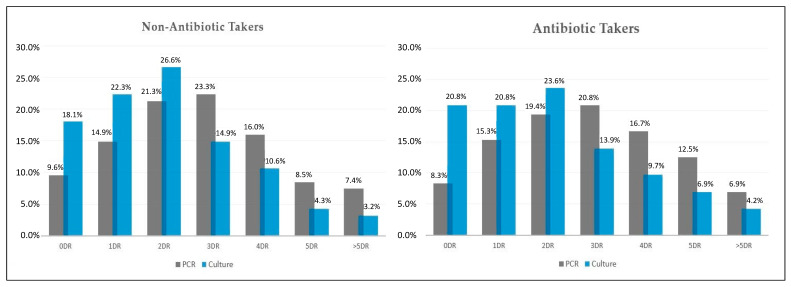
Parallel results of antimicrobial drug resistance pattern in non-antibiotic takers (*n* = 94) and antibiotic takers (*n* = 72), identified by multiplex PCR and conventional bacterial cultures. DR—drug resistance.

As presented in Figure 3, the parallel comparison of antimicrobial drug resistance determined by multiplex PCR test and conventional bacterial culture in COVID-19 patients with secondary bacterial infection, identified a significant difference in proportions. In the group of antibiotic takers, bacterial cultures failed to identify 12.5% of drug resistant samples (8.3% identified by PCR vs. 20.8% identified by culture). The PCR method identified 36.1% of samples resistant to more than three antimicrobials, compared with 20.8% identified in bacterial cultures (Figure 3). In non-antibiotic takers, a total of 31.9% of samples tested by PCR were resistant to more than three antimicrobials. Out of these samples, bacterial cultures identified only 18.1% as resistant to more than three antimicrobials (Figure 3).

A comparison of COVID-19 patient outcomes determined by the status of antibiotic use and the first bacterial identification test performed after hospital admission is presented in Table 5. A statistically significant difference between multiplex PCR and conventional cultures was observed in regard to the time taken until test confirmation, in both groups of antibiotic takers and non-antibiotic takers (PCR test = 13.4 h vs. culture = 25.1 h, *p*-value < 0.001), respectively (PCR test = 12.9 h vs. culture = 24.7 h, *p*-value < 0.001). The duration of time from hospital admission until therapeutic antimicrobial treatment was initiated also differed significantly between both study groups by the test used for bacterial identification (PCR test = 28.8 h vs. culture = 40.4 h, *p*-value < 0.001), respectively (PCR test = 25.3 h vs. culture = 41.6 h, *p*-value < 0.001). Lastly, COVID-19 patients self-medicating with antibiotics that had an initial assessment for secondary bacterial infection by multiplex PCR spent 12.5 days in the hospital, compared with 14.9 days spent by those who had an initial test for bacterial identification using a conventional culture method (*p*-value = 0.004). Non-antibiotic takers that had an initial assessment for secondary bacterial infection by multiplex PCR spent 12.0 days in the hospital, compared with 14.5 days spent by those who had an initial test for bacterial identification using a conventional culture method (*p*-value < 0.001).

**Figure 4:**
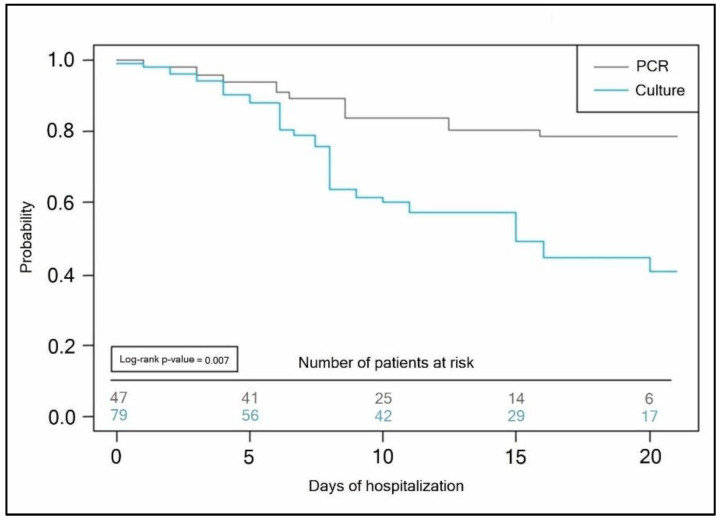
Kaplan–Meyer probability curve of hospitalization duration by the method of bacterial identification test employed in patients self-medicating with antibiotics.

**Figure 5:**
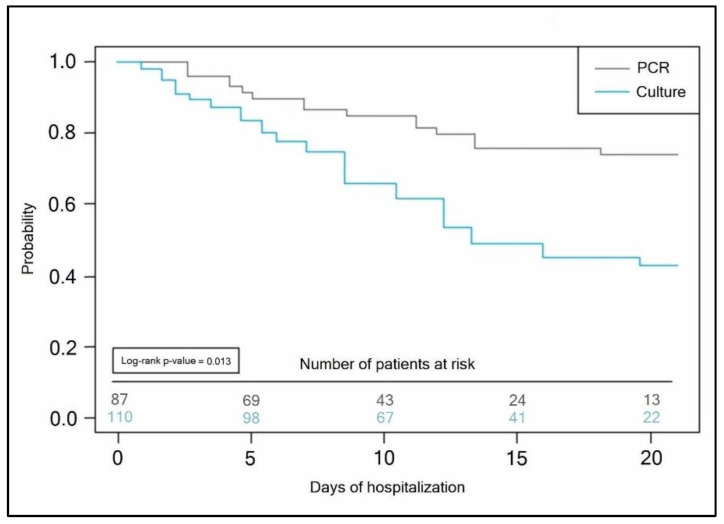
Kaplan–Meyer probability curve of hospitalization duration by the method of bacterial identification test employed in non-antibiotic takers.

The Kaplan–Meyer tests presented in Figure 4 and Figure 5 predicted the probability of a longer hospital stay for COVID-19 patients with secondary bacterial infection when the initial bacterial identification test was a conventional bacterial culture (antibiotic takers log-rank *p*-value = 0.007, vs. non-antibiotic takers log-rank *p*-value = 0.013).

The risk factor analysis presented in Table 6 was adjusted for age, COVID-19 severity, and pulmonary disease. It was observed that over-the-counter use of antibiotics represented a significant independent risk factor for a prolonged hospitalization (AOR = 1.21, *p*-value = 0.042). Similar findings were observed for smoking status (AOR = 1.44, *p*-value < 0.001), bacterial superinfection (AOR = 1.52, *p*-value < 0.001), performing only a conventional bacterial culture (AOR = 1.17, *p*-value = 0.009), and a duration for bacterial sampling longer than 48 h from hospital admission (AOR = 1.36, *p*-value = 0.001).

## 3. Discussion

In this study, bacterial superinfections were the most common patient presentation. It was observed that COVID-19 patients self-medicated with antibiotics were more likely to have a superinfection in 63.6% of the hospital admissions for secondary bacterial infections. Among the participants, almost one-third (32.2%) had asked their doctor to prescribe antibiotics for an illness, and this request was significantly related to taking antibiotics for COVID-19. Probably the most common misunderstanding these patients have is that antibiotics can treat the common cold and generally all respiratory infections such as COVID-19. As bacterial cultures often return false-negative results in samples provided by antibiotic takers, the multiplex PCR analysis proved to be a more accurate tool for bacterial identification and a more efficient method of testing, which reduced the duration of hospital stay by an average of 2.5 days.

The discrepancy between the results obtained from culture experiments and those derived from a PCR molecular analysis is most likely due to antibiotic use, although the multiplex PCR showed better accuracy even in non-antibiotic takers. PCR targeting the 16S rRNA gene has been shown to effectively detect and identify living or dead bacteria in patients receiving antibiotic treatment and in symptomatic patients with a culture-negative microbiological report [23,24]. Microbiological cultures are often negative in symptomatic infections in different body locations, from which samples are acquired that are positive for PCR targeting bacterial DNA. The involvement of injured, starved, and viable but nonculturable bacteria in cases of infection with culture-negative, PCR-positive reports has been suggested because these microbial forms are rarely or never recoverable in culture media, can represent survival strategies that are activated by antibiotic treatment, and appear to retain their pathogenic potential.

Other investigations show a considerable proportion of culture-negative samples containing bacterial DNA (14%), which is often found using not only a universal pair of primers but also primers targeting particular bacteria. When only individuals with symptomatic illnesses were evaluated, this proportion of culture-negative, PCR-positive samples increased to 27% [25,26]. Recent research compared the quantitative bacterial culture with the molecular PCR method in evaluating microbiologic data in association with antibiotic medication, where parallel results showed disparities. Although the accuracy of the PCR was higher, and identified more false negatives and false positives than bacterial cultures [27], the researchers did not correlate their results with patient outcomes and the clinical impact of using a more accurate and faster diagnosis method, as determined in our study. On the other hand, a larger study that analyzed 1370 samples concluded that the PCR method significantly boosts the rate of pathogen identification, although parallel cultivation is necessary, because of the possibility of false-negative PCR findings [28].

This study identified *Staphylococcus aureus*, *Klebsiella spp*., and *Pseudomonas aeruginosa*, as the most common bacteria involved in secondary infections in COVID-19 patients. COVID-19 is caused by an extremely contagious infectious pathogen that spreads mostly by droplets and intimate contact. Numerous family infection clusters have been identified, and some of the confirmed cases occurred in hospitals. In a recent study from Belgium, 40 percent of patients had coinfection with *Staphylococcus aureus*, *Haemophilus influenzae* and *Moraxella catarrhalis* as the most often found bacteria with multiple genome copies. In Italy, the frequency of *Haemophilus influenzae* infections was 56% among COVID-19 patients [29]. However, one German study revealed that the occurrence of bacterial coinfections was only 34% [30], while other more recent investigations showed that the majority of patients were coinfected with several other bacteria such as *Klebsiella spp*. and *Escherichia coli* [31], rather than *Haemophilus influenzae*. These differences might be attributed to hospital conditions that vary by region and economy and also by the SARS-CoV-2 strain involved.

Antibiotic self-medication is a significant contributor to antimicrobial resistance. One of the study limitations is that only patients hospitalized were assessed, while the number of those self-medicating with antibiotics at home might be much higher. It was previously determined in an Australian study that during the first pandemic wave, 19.5% of the participants used antibiotics to protect themselves against COVID-19 [32]. Participants who received antibiotics for COVID-19 reported considerably more psychological suffering than those who did not receive antibiotics. In the same study, antibiotic awareness was shown to be fairly prevalent among the Australian people, as probably happens in the Romanian population, and in certain cases, ignorance was found to be strongly related to the use of antibiotics for COVID-19. Moreover, a recent systematic review determined that the prevalence of self-medication with antibiotics ranged from 4% to 88% [33]. Another study limitation is the significantly higher prevalence of chronic respiratory diseases in the group of COVID-19 patients self-medicating with antibiotics, implying that some results may be biased. Lastly, recall bias is an important limitation of the study, since study participants showed inconsistent responses to the question about the duration and dose of antibiotic self-administration.

## 4. Materials and Methods

### 4.1. Study Design and Ethical Considerations

This multicenter research was designed as a cross-sectional study of hospitalized patients with COVID-19 and secondary bacterial infections who self-medicated with antibiotics. The setting comprised two tertiary hospitals in Western Romania, where patients were admitted to the COVID-19 units of the Internal Medicine Department of Timisoara Municipal Emergency Hospital and the Infectious Diseases and Pulmonology Hospital, “Victor Babes” University in the period starting January 2020 until January 2022. The research protocol was approved by the Ethics Committee of the “Victor Babes” University of Medicine and Pharmacy in Timisoara, Romania, and by the Ethics Committee of both hospitals.

A search of the database and patients’ paper records was conducted to determine the cases of bacterial coinfection and superinfection in patients with COVID-19 from the two hospitals. Patients who confirmed taking antibiotics at home since the onset of COVID-19 symptoms were included in the “Antibiotic Takers” group, while those who did not take antibiotics before admission were defined as “Non-Antibiotic Takers”. The duration of the self-medicated antibiotic treatment was assumed to be the same as the number of days elapsed from symptom onset until hospital admission, since the recall ability of patients was inconsistent and most of them decided to take antibiotics close to initial symptom onset. A further database and paper records search was necessary to determine the type of bacterial identification test performed initially to diagnose the secondary bacterial infection, either by a multiplex RT-PCR test or a conventional bacterial culture.

### 4.2. Inclusion Criteria and Study Variables

We used a convenience sampling approach to determine the ideal sample size, which was determined to be at least 385 patients, for a 5% margin of error at a 95% level of confidence. The inclusion criteria were set for all patients over 18 years old with a history of hospital admission for SARS-CoV-2 infection diagnosed by real-time polymerase chain reaction (RT-PCR) in the two hospital departments involved in the current study. All patients were included if their records mentioned a secondary bacterial infection diagnosed during their hospital stay. Patients with immune deficiencies and immune-suppressive therapy were excluded from the study. A secondary bacterial infection was considered to be either a coinfection or a superinfection. Coinfections were defined as those cases diagnosed during the first 48 h of COVID-19 hospitalization, and superinfections as cases diagnosed after 48 h of hospitalization.

The variables considered important for evaluation were background data (age, sex, body mass index, antibiotic consumption behavior, smoking status, history of pulmonary disease, and other existing comorbidities), patient outcomes, including the number of days from symptom onset until hospitalization, COVID-19 complications, type of secondary bacterial infection (coinfection/superinfection), tests performed for bacterial identification (multiplex PCR or bacterial culture), pulmonary ground-glass opacities, COVID-19 severity, oxygen supplementation (AIRVO, CPAP, ventilator), ICU admission, duration of ICU admission, the number of days elapsed between first COVID-19 symptoms until death, mortality, and duration of hospital stay. Biological parameters that were assessed included the complete blood count (red blood cells, platelets, white blood cells, hemoglobin, hematocrit), kidney function tests (creatinine, blood urea nitrogen, glomerular filtration rate), liver function tests (alanine aminotransferase, aspartate aminotransferase, gamma glutamyl transpeptidase, prothrombin time), and inflammatory markers (procalcitonin, c-reactive protein, interleukin-6, erythrocyte sedimentation rate, fibrinogen, D-dimers). For microbial identification, we considered the number of positive specimens were counted (sputum/aspirate, blood, urine, and feces), the pathogens involved, the percentage of antibiotic resistance given by PCR or culture tests, the percentage of multidrug resistance, the number of pathogens identified, time of sampling (before or after 48 h since admission), numbers of specimens taken, the time elapsed from sampling until results were given, the time elapsed from hospital admission until therapeutic antibiotic initiation, and lastly, the percentage of discontinued, changed, or continued antibiotics.

### 4.3. Materials Used

The fluid and solid samples were taken from COVID-19 patients for analysis according to standard microbiological methods at the Microbiology Unit of the two clinics involved in the study. The standard microbiological method included microscopic observation of the sample after Gram staining and the inoculation of the sample in growing media (conventional plate culture or blood culture). The conventional plate culture is a qualitative and quantitative technique that relies on the sample being deposited in or on an agar layer in a Petri dish. Individual organisms or small groups of organisms occupy a defined spot in the agar and develop to form discrete colonies that are visually counted during incubation. Numerous varieties of agar medium may be used in this manner to grow and count various bacteria, as well as to test for antibiotic susceptibility [34,35]. The PCR multiplex protocol was carried out with 1 mL of bodily fluid that was centrifuged for 5 minutes to extract DNA, and total DNA was recovered from the pellet, according to existing guidelines [36]. PCR experiments were conducted using a conventional PCR technique on a Gene Amp PCR System 9700 thermal cycler (Perkin-Elmer), and amplification products were purified using the QIAEX II Gel Extraction Kit (Qiagen) and evaluated by sequencing (BMR Genomics, Padua, Italy).

### 4.4. Statistical Analysis

The statistical analysis was performed with IBM SPSS v.26 and MedCalc v.20. The absolute and relative frequencies of categorical variables were computed. For a comparison of the proportions, the Chi-square and Fisher’s tests were employed, while for the comparison of group differences in nonparametric data, the Mann–Whitney test was used. Parametric continuous variables that followed a normal distribution were compared by mean and standard deviation with the Student’s *t*-test (unpaired, independent samples). A Kaplan–Meyer curve was plotted for the probabilities of the duration of hospital stay by the type of bacterial identification test employed. Lastly, a multivariate analysis adjusted for confounding factors was performed to determine the independent risk factors for prolonged hospital stay in COVID-19 patients with secondary bacterial infections who self-medicated with antibiotics. The significance threshold was set for an alpha value of 0.05.

## 5. Conclusions

The multiplex PCR tool can be a very efficient diagnostic tool for secondary bacterial infections in COVID-19 patients who self-medicate with antibiotics. Using this method as an initial screening in COVID-19 patients that show signs of sepsis and clinical deterioration will help to shorten recovery time and hospital admission. We have come to appreciate the critical nature of prudent antibiotic usage, particularly during the COVID period, to avoid unneeded antibiotic treatments and halt the emergence of antimicrobial resistance. Additionally, we propose shortening the turnaround time for tests in the diagnostic route for COVID-19 patients in order to reduce the need to take antibiotics, and we argue for the inclusion of antimicrobial stewardship initiatives in the pandemic response.

## Figures and Tables

**Table 1:** General characteristics of COVID-19 patients with secondary bacterial infections stratified by antibiotic-use behavior.

Variables *	Antibiotic Takers (*n* = 198)	Non-Antibiotic Takers (*n* = 291)	*p*-Value
**Age**			0.632
18–40 years	28 (14.2%)	49 (16.8%)	
40–65 years	95 (47.9%)	129 (44.3%)	
>65 years	75 (37.9%)	113 (38.8%)	
Sex			0.177
Men	117 (49.1%)	154 (52.9%)	
Women	81 (40.9%)	137 (47.1%)	
**BMI**			
Underweight (<18.5 kg/m^2^)	14 (7.1%)	23 (7.9%)	0.923
Normal weight (18.5–25.0 kg/m^2^)	106 (53.5%)	157 (53.9%)	
Overweight (>25.0 kg/m^2^)	78 (39.4%)	111 (38.2%)	
**Antibiotic consumption behavior**			-
By prescription	65 (32.8%)	-	
Over-the-counter	133 (67.2%)	-	
**Smoking status**			0.292
Yes	63 (31.8%)	106 (36.4%)	
No	135 (68.2%)	185 (63.6%)	
**Pulmonary disease**			
Chronic bronchitis	47 (23.7%)	39 (13.4%)	0.003
COPD	24 (12.1%)	17 (5.8%)	0.013
Asthma	19 (9.6%)	16 (5.5%)	0.084
Pulmonary hypertension	2 (1.0%)	1 (0.3%)	0.354
Lung cancer	2 (1.0%)	4 (1.4%)	0.719
**Other comorbidities**			
Cardiac	64 (32.3%)	98 (33.6%)	0.754
Metabolic	37 (18.7%)	46 (15.8%)	0.405
Cerebrovascular	12 (6.1%)	17 (5.8%)	0.919
Digestive & liver	16 (8.1%)	20 (10.1%)	0.615
Kidney disease	13 (6.6%)	19 (6.5%)	0.987
Malignancy **	4 (2.0%)	4 (1.4%)	0.580

* Data reported as *n* (%), and calculated using Chi-square test and Fisher’s exact unless specified differently; ** Excluding lung cancer; BMI—Body Mass Index; COPD—Chronic Obstructive Pulmonary Disease.

**Table 2:** Outcomes of COVID-19 patients with secondary bacterial infections stratified by antibiotic use behavior.

Variables *	Antibiotic Takers (*n* = 198)	Non-Antibiotic Takers (*n* = 291)	*p*-Value
Days from symptom onset until hospitalization, (mean ± SD)	4.2 ± 1.5	4.0 ± 1.4	0.132 *^t^*
**Complications**			
ARDS	16 (8.1%)	19 (6.5%)	0.513
Ventilator-associated pneumonia	12 (6.1%)	7 (2.4%)	0.040
Community-acquired pneumonia	29 (14.6%)	32 (10.9%)	0.230
Asthma exacerbation	9 (4.5%)	8 (2.7%)	0.292
COPD exacerbation	7 (3.5%)	11 (3.7%)	0.887
**Secondary bacterial infection**			0.015
Bacterial coinfection (<48 h)	72 (36.4%)	138 (47.4%)	
Bacterial superinfection (>48 h)	126 (63.6%)	153 (52.6%)	
**Performed tests**			0.310
Culture	79 (39.9%)	110 (37.8%)	
PCR	47 (23.7%)	87 (29.9%)	
Culture and PCR	72 (36.4%)	94 (32.3%)	
**Ground glass opacities**			0.022
<30%	63 (31.8%)	123 (42.3%)	
30–60%	113 (57.1%)	150 (51.5%)	
>60%	22 (11.1%)	18 (6.2%)	0.047
**COVID-19 severity**			
Mild	68 (34.3%)	127 (43.6%)	
Moderate	106 (53.5%)	143 (49.1%)	
Severe	24 (12.1%)	21 (7.2%)	
**Oxygen supplementation**			
AIRVO	86 (43.4%)	108 (37.1%)	0.160
CPAP	24 (12.1%)	31 (10.7%)	0.613
Ventilator	21 (10.6%)	15 (5.6%)	0.023
**Outcomes**			
ICU admission	19 (9.6%)	14 (4.8%)	0.038
Days in the ICU (mean ± SD)	12.9 ± 6.5	11.6 ± 5.2	0.014 *^t^*
Days between symptom onset until death (mean ± SD)	15.2 ± 6.6	13.7 ± 6.0	0.009 *^t^*
Mortality	14 (7.1%)	13 (4.5%)	0.215
Days until discharge (mean ± SD)	12.8 ± 4.6	12.0 ± 5.1	0.077 *^t^*

* Data reported as *n* (%), and calculated using Chi-square test and Fisher’s exact unless specified differently; *^t^*—Unpaired Student’s *t*-test; SD—Standard Deviation; ARDS—Acute Respiratory Distress Syndrome; COPD—Chronic Obstructive Pulmonary Disease; PCR—Polymerase Chain Reaction; AIRVO—Noninvasive high-flow nasal oxygen therapy; CPAP—Continuous Positive Airway pressure; ICU—Intensive Care Unit.

**Table 3:** Biological parameters at admission of COVID-19 patients with secondary bacterial infections stratified by antibiotic use behavior.

Variables *	Normal Range	Antibiotic Takers (*n* = 198)	Non-Antibiotic Takers (*n* = 291)	*p*-Value
**Complete blood count**				
RBC (millions/mm^3^)	4.35–5.65	4.38 ± 1.1	4.41 ± 1.3	0.790
PLT (thousands/mm^3^)	150–450	186 ± 53	195 ± 61	0.092
WBC (thousands/mm^3^)	4.5–11.0	15.2 ± 6.0	14.7 ± 5.6	0.347
Hb (g/dL)	13.0–17.0	13.6 ± 2.2	14.0 ± 2.4	0.062
Hematocrit (%)	36–48	37 ± 7	38 ± 8	0.154
**Kidney function tests**				
Creatinine (µmol/L)	0.74–1.35	1.36 ± 0.33	1.28 ± 0.29	0.004
BUN (mmol/L)	2.1–8.5	9.1 ± 3.4	8.6 ± 2.2	0.048
GFR	>60	74 ± 12	76 ± 13	0.085
**Liver function tests**				
ALT (U/L)	7–35	53 ± 16	49 ± 18	0.012
AST (U/L)	10–40	44 ± 9	42 ± 9	0.016
GGT (U/L)	0–30	14.6 ± 4	15.1 ± 4	0175
PT (seconds)	11.0–13.5	11.8 ± 1.5	11.9 ± 1.7	0.503
**Inflammatory markers** **				
Procalcitonin (ug/L)	0–0.25 ug/L	0.7 [0.2–1.0]	0.6 [0.1–0.9]	0.264
CRP (mg/L)	0–10 mg/L	34 [12–49]	32 [13–47]	0.139
IL-6 (pg/mL)	0–16 pg/mL	42 [28–49]	37 [24–45]	0.016
ESR (mm/h)	0–22 mm/hr	43 [36–54]	41 [35–52]	0.088
Fibrinogen (g/L)	2–4 g/L	5.1 [3.8–6.6]	4.5 [3.4–5.7]	0.003
D-dimer (ng/mL)	<250	361 [308–442]	372 [311–436]	0.063

* Data reported as mean ± SD and compared by independent samples *t*-test, unless specified differently; ** Data reported as median [IQR] and compared by Mann–Whitney U-test; RBC—Red Blood Cells; PLT—Platelets; WBC—White Blood Cells; Hb—Hemoglobin; BUN—Blood Urea Nitrogen; GFR—Glomerular filtration Rate; CRP—C-reactive Protein; IL—Interleukin; ESR—Erythrocyte Sedimentation Rate.

**Table 4:** Parallel comparison of bacterial identification test results in patients with COVID-19 stratified by antibiotic use.

Variables *	Antibiotic Takers		*p*-Value **	Non-Antibiotic Takers		*p*-Value **
	PCR (*n* = 72)	Culture (*n* = 72)		PCR (*n* = 94)	Culture (*n* = 94)	
**Positive specimens**						
Sputum/Aspirate	66/72 (91.7%)	37/72 (51.4%)	<0.001	88/94 (93.6%)	81/94 (86.2%)	0.090
Blood	62/72 (86.1%)	41/72 (56.9%)	<0.001	83/94 (88.3%)	74/94 (78.7%)	0.076
Urine	18/24 (75.0%)	10/24 (41.7%)	0.019	49/60 (71.7%)	53/60 (65.0%)	0.297
Fecal	5/7 (71.4%)	3/7 (42.9%)	0.280	6/9 (66.7%)	5/9 (55.6%)	0.550
False negative result	24/175 (13.7%)	84/175 (48.0%)	<0.001	31/257 (12.1%)	44/257 (17.1%)	0.104
**Pathogens involved**						
*Staphylococcus aureus*	16 (22.2%)	7 (9.7%)	0.040	21 (22.3%)	16 (17.0%)	0.359
*Streptococcus pneumoniae*	6 (8.3%)	4 (5.6%)	0.512	15 (16.0%)	7 (7.4%)	0.069
*Streptococcus pyogenes*	10 (13.9%)	5 (6.9%)	0.172	8 (8.5%)	5 (5.3%)	0.388
*Enterococcus faecalis*	5 (6.9%)	2 (2.8%)	0.245	7 (7.4%)	3 (3.2%)	0.193
*Escherichia coli*	14 (19.4%)	8 (11.1%)	0.164	19 (20.2%)	9 (9.6%)	0.040
*Klebsiella spp*	18 (25.0%)	9 (12.5%)	0.054	26 (27.7%)	14 (14.9%)	0.032
*Pseudomonas aeruginosa*	20 (27.8%)	9 (12.5%)	0.022	17 (18.1%)	10 (10.6%)	0.145
*Clostridium difficile*	5 (6.9%)	3 (4.2%)	0.466	9 (9.6%)	6 (6.4%)	0.419
Others	7 (9.7%)	2 (2.8%)	0.085	6 (6.4%)	2 (2.1%)	0.148
**Antibiotic resistance percentage**						
Cephalosporin	31 (43.1%)	19 (26.4%)	0.035	29 (30.9%)	20 (21.3%)	0.134
Macrolide	28 (38.9%)	12 (16.7%)	0.002	26 (27.7%)	21 (22.3%)	0.399
Penicillin	55 (76.4%)	48 (66.7%)	0.196	53 (56.4%)	41 (43.6%)	0.080
Aminoglycoside	23 (31.9%)	19 (26.4%)	0.463	20 (21.3%)	17 (18.1%)	0.582
Tetracycline	19 (26.4%)	14 (19.4%)	0.321	21 (22.3%)	14 (14.9%)	0.189
Quinolones	18 (25.0%)	10 (13.9%)	0.092	15 (16.0%)	11 (11.7%)	0.389
Carbapenems	8 (11.1%)	7 (9.7%)	0.785	13 (13.8%)	10 (10.6%)	0.504
Glycopeptides	19 (26.4%)	10 (13.9%)	0.061	11 (11.7%)	6 (6.4%)	0.203
Nitroimidazole	9 (12.5%)	7 (9.7%)	0.595	9 (9.6%)	4 (4.3%)	0.150
Other	7 (9.7%)	6 (8.3%)	0.771	9 (9.6%)	3 (3.2%)	0.073
**Multidrug resistance**			0.033			0.091
Yes	(91.7%)	(79.2%)		85 (90.4%)	77 (81.9%)	
No	(8.3%)	(20.8%)		9 (9.6%)	17 (18.1%)	
**Number of pathogens identified**						
Monoinfection	43 (59.7%)	30 (41.7%)	0.030	60 (63.8%)	52 (55.3%)	0.235
Two pathogens	21 (29.2%)	7 (9.7%)	0.003	26 (27.7%)	17 (18.1%)	0.118
More than two pathogens	8 (11.1%)	2 (2.8%)	0.049	8 (8.5%)	3 (3.2%)	0.120

* Data reported as *n* (%) unless specified differently; ** Chi-square test and Fisher’s exact.

**Table 5:** Comparison of COVID-19 patient outcomes stratified by antibiotic use and type of bacterial identification test performed.

Variables *	Antibiotic Takers		*p*-Value	Non-Antibiotic Takers		*p*-Value **
	PCR (*n* = 47)	Culture (*n* = 79)		PCR (*n* = 87)	Culture (*n* = 110)	
**Time of sampling**			0.097			0.272
Within 48 h from admission	28 (59.6%)	35 (44.3%)		43 (49.4%)	63 (57.3%)	
After 48 h from admission	19 (40.4%)	44 (55.7%)		44 (50.6%)	47 (42.7%)	
**Specimens taken**						
Sputum/Aspirate	41 (87.2%)	73 (92.4%)	0.338	82 (94.3%)	104 (94.5%)	0.929
Blood	38 (80.9%)	66 (83.5%)	0.700	74 (85.1%)	95 (86.4%)	0.794
Urine	15 (31.9%)	19 (24.1%)	0.336	25 (28.7%)	22 (20.0%)	0.153
Fecal	7 (14.9%)	22 (27.8%)	0.094	13 (14.9%)	15 (13.6%)	0.794
**Timeline**						
Time to results, hours (mean ± SD)	13.4 ± 3.5	25.1 ± 4.9	<0.001 *^t^*	12.9 ± 4.2	24.7 ± 4.7	<0.001 *^t^*
Time from admission to therapeutic antibiotic initiation, hours (mean ± SD)	26.8 ± 7.5	40.4 ± 11.4	<0.001 *^t^*	25.3 ± 7.0	41.6 ± 7.2	<0.001 *^t^*
**Decision**			0.743			0.574
Discontinued antibiotics	6 (12.8%)	11 (13.9%)		13 (14.9%)	12 (10.9%)	
Changed antibiotic	38 (80.9%)	60 (75.9%)		65 (74.7%)	89 (80.9%)	
Continued antibiotic	3 (6.4%)	8 (10.1%)		9 (10.3%)	9 (8.2%)	
Days until discharge (mean ± SD)	12.4 ± 4.3	14.9 ± 4.8	0.004 *^t^*	12.0 ± 4.1	14.5 ± 4.3	<0.001 ^*t*^

* Data reported as *n* (%) unless specified differently; ** Chi-square test and Fisher’s exact, unless specified differently; *^t^*—Unpaired Student’s *t*-test; SD—Standard Deviation.

**Table 6:** Risk factor analysis for prolonged hospitalization in COVID-19 patients with secondary bacterial infections self-medicated with antibiotics.

Factors *	Adjusted OR	95% CI	*p*-Value
**Antibiotic consumption behavior**			
By prescription ^^^	1.04	0.87–1.21	0.296
Over-the-counter	1.21	1.02–1.34	0.042
**Smoking status**			
No	0.93	0.71–1.05	0.137
Yes ^^^	1.44	1.12–1.69	<0.001
**Secondary bacterial infection**			
Bacterial coinfection (<48 h) ^^^	1.09	0.94–1.15	0.058
Bacterial superinfection (>48 h)	1.52	1.38–1.93	<0.001
**Performed tests**			
Culture	1.17	1.01–1.49	0.009
PCR	0.98	0.82–1.14	0.221
Culture and PCR ^^^	0.92	0.77–1.09	0.375
**Time of sampling**			
Within 48 h from admission ^^^	1.02	0.93–1.22	0.072
After 48 h from admission	1.36	1.04–1.78	0.001

^^^ Reference category; * Adjusted by age, COVID-19 severity, and pulmonary diseases.

## Data Availability

Data available on request.

## References

[1] Şahin Ş., Boado-Penas M.d.C., Constantinescu C., Eisenberg J., Henshaw K., Hu M., Wang J., Zhu W. (2020). First Quarter Chronicle of COVID-19: An Attempt to Measure Governments’ Responses. Risks.

[2] Karlafti E., Anagnostis A., Kotzakioulafi E., Vittoraki M.C., Eufraimidou A., Kasarjyan K., Eufraimidou K., Dimitriadou G., Kakanis C., Anthopoulos M. (2021). Does COVID-19 Clinical Status Associate with Outcome Severity? An Unsupervised Machine Learning Approach for Knowledge Ex-traction. J. Pers. Med..

[3] Marincu I., Bratosin F., Vidican I., Bostanaru A.-C., Frent S., Cerbu B., Turaiche M., Tirnea L., Timircan M. (2021). Predictive Value of Comorbid Conditions for COVID-19 Mortality. J. Clin. Med..

[4] Pérez-Lazo G., Silva-Caso W., del Valle-Mendoza J., Morales-Moreno A., Ballena-López J., Soto-Febres F., Martins-Luna J., Carrillo-Ng H., del Valle L.J., Kym S. (2021). Identification of Coinfections by Viral and Bacterial Pathogens in COVID-19 Hospitalized Patients in Peru: Molecular Diagnosis and Clinical Characteristics. Antibiotics.

[5] Uyeki T.M., Bernstein H.H., Bradley J.S., Englund J.A., File T.M., Fry A.M., Gravenstein S., Hayden F.G., Harper S.A., Hirshon J.M. (2019). Clinical Practice Guidelines by the Infectious Diseases Society of America: 2018 Update on Diagnosis, Treatment, Chemoprophylaxis, and Institutional Outbreak Management of Seasonal Influenzaa. Clin. Infect. Dis..

[6] Klein E.Y., Monteforte B., Gupta A., Jiang W., May L., Hsieh Y., Dugas A. (2016). The frequency of influenza and bacterial coinfection: A systematic review and meta-analysis. Influ. Other Respir. Viruses.

[7] Kim D., Quinn J., Pinsky B., Shah N.H., Brown I. (2020). Rates of coinfection between SARS-CoV-2 and other respiratory pathogens. Jama.

[8] Alhumaid S., Al Mutair A., Al Alawi Z., Alshawi A.M., Alomran S.A., Almuhanna M.S., Almuslim A.A., Bu Shafia A.H., Alotaibi A.M., Ahmed G.Y. (2021). Coinfections with Bacteria, Fungi, and Respiratory Viruses in Patients with SARS-CoV-2: A Systematic Review and Meta-Analysis. Pathogens.

[9] Oliva J., Terrier O. (2021). Viral and Bacterial Co-Infections in the Lungs: Dangerous Liaisons. Viruses.

[10] Langford B.J., So M., Raybardhan S., Leung V., Westwood D., MacFadden D.R., Soucy J.-P.R., Daneman N. (2020). Bacterial coinfection and secondary infection in patients with COVID-19: A living rapid review and meta-analysis. Clin. Microbiol. Infect..

[11] Cohen R., Babushkin F., Finn T., Geller K., Alexander H., Datnow C., Uda M., Shapiro M., Paikin S., Lellouche J. (2021). High Rates of Bacterial Pulmonary Co-Infections and Superinfections Identified by Multiplex PCR among Critically Ill COVID-19 Patients. Microorganisms.

[12] Maes M., Higginson E., Pereira-Dias J., Curran M.D., Parmar S., Khokhar F., Cuchet-Lourenço D., Lux J., Sharma-Hajela S., Ravenhill B. (2021). Ventilator-associated pneumonia in critically ill patients with COVID-19. Crit. Care.

[13] Rouyer M., Strazzulla A., Youbong T., Tarteret P., Pitsch A., de Pontfarcy A., Cassard B., Vignier N., Pourcine F., Jochmans S. (2021). Ven-tilator-Associated Pneumonia in COVID-19 Patients: A Retrospective Cohort Study. Antibiotics.

[14] Sreenath K., Batra P., Vinayaraj E.V., Bhatia R., SaiKiran K., Singh V., Singh S., Verma N., Singh U.B., Mohan A. (2021). Coinfections with Other Respiratory Pathogens among Patients with COVID-19. Microbiol Spectr..

[15] Feldman C., Anderson R. (2021). The role of coinfections and secondary infections in patients with COVID-19. Pneumonia.

[16] Carvalho-Pereira J., Fernandes F., Araújo R., Springer J., Loeffler J., Buitrago M.J., Pais C., Sampaio P. (2020). Multiplex PCR Based Strategy for Detection of Fungal Pathogen DNA in Patients with Suspected Invasive Fungal Infections. J. Fungi.

[17] Arrivé F., Coudroy R., Thille A.W. (2021). Early Identification and Diagnostic Approach in Acute Respiratory Distress Syndrome (ARDS). Diagnostics.

[18] Martin-Loeches I., Motos A., Menéndez R., Gabarrús A., González J., Fernández-Barat L., Ceccato A., Pérez-Arnal R., García-Gasulla D., Ferrer R. (2022). ICU-Acquired Pneumonia Is Associated with Poor Health Post-COVID-19 Syndrome. J. Clin. Med..

[19] Qasem A., Shaw A.M., Elkamel E., Naser S.A. (2021). Coronavirus Disease 2019 (COVID-19) Diagnostic Tools: A Focus on Detection Technologies and Limitations. Curr. Issues Mol. Biol..

[20] Murray C.J., Ikuta K.S., Sharara F., Swetschinski L., Aguilar G.R., Gray A., Han C., Bisignano C., Rao P., Wool E. (2022). Global burden of bacterial antimicrobial resistance in 2019: A systematic analysis. Lancet.

[21] Aslam A., Gajdács M., Zin C.S., Rahman N.S.B.A., Ahmed S.I., Jamshed S.Q. (2020). Public Awareness and Practices towards Self-Medication with Antibiotics among the Malaysian Population. A Development of Questionnaire and Pilot-Testing. Antibiotics.

[22] Kalam M.A., Shano S., Afrose S., Uddin M.N., Rahman N., Jalal F.A., Akter S., Islam A., Anam M.M., Hassan M.M. (2022). Antibiotics in the Community During the COVID-19 Pandemic: A Qualitative Study to Understand Users’ Perspectives of Antibiotic Seeking and Consumption Behaviors in Bangladesh. Patient Prefer Adher..

[23] Rogers G.B., Russell L.E., Preston P.G., Marsh P., Collins J.E., Saunders J., Sutton J., Fine D., Bruce K.D., Wright M. (2010). Characterisation of bacteria in ascites–reporting the potential of culture-independent, molecular analysis. Eur. J. Clin. Microbiol. Infect. Dis..

[24] Lardaro T., Wang A.Z., Bucca A., Croft A., Glober N., Holt D.B., Musey P.I., Peterson K.D., Trigonis R.A., Schaffer J.T. (2021). Characteristics of COVID-19 patients with bacterial coinfection admitted to the hospital from the emergency department in a large regional healthcare system. J. Med. Virol..

[25] Lleo M.M., Ghidini V., Tafi M.C., Castellani F., Trento I., Boaretti M. (2014). Detecting the presence of bacterial DNA by PCR can be useful in diagnosing culture-negative cases of infection, especially in patients with suspected infection and antibiotic therapy. FEMS Microbiol. Lett..

[26] Cohen R., Finn T., Babushkin F., Geller K., Alexander H., Shapiro M., Uda M., Mostrchy A.R., Amash R., Shimoni Z. (2022). High rate of bacterial respiratory tract coinfections upon admission amongst moderate to severe COVID-19 patients. Infect. Dis..

[27] Collins M.E., Popowitch E.B., Miller M.B. (2020). Evaluation of a Novel Multiplex PCR Panel Compared to Quantitative Bacterial Culture for Diagnosis of Lower Respiratory Tract Infections. J. Clin. Microbiol..

[28] Tkadlec J., Peckova M., Sramkova L., Rohn V., Jahoda D., Raszka D., Berousek J., Mosna F., Vymazal T., Kvapil M. (2019). The use of broad-range bacterial PCR in the diagnosis of infectious diseases: A prospective cohort study. Clin. Microbiol. Infect..

[29] Baccolini V., Migliara G., Isonne C., Dorelli B., Barone L., Giannini D., Marotta D., Marte M., Mazzalai E., Alessandri F. (2021). The impact of the COVID-19 pandemic on healthcare-associated infections in intensive care unit patients: A retrospective cohort study. Antimicrob. Resist. Infect. Control.

[30] Rothe K., Feihl S., Schneider J., Wallnöfer F., Wurst M., Lukas M., Treiber M., Lahmer T., Heim M., Dommasch M. (2021). Rates of bacterial coinfections and an-timicrobial use in COVID-19 patients: A retrospective cohort study in light of antibiotic stewardship. Eur. J. Clin. Microbiol. Infect. Dis..

[31] Ahmed N., Khan M., Saleem W., Karobari M.I., Mohamed R.N., Heboyan A., Rabaan A.A., Mutair A.A., Alhumaid S., Alsadiq S.A. (2022). Evaluation of Bi-Lateral Co-Infections and Antibiotic Resistance Rates among COVID-19 Patients. Antibiotics.

[32] Zhang A., Hobman E.V., De Barro P., Young A., Carter D.J., Byrne M. (2021). Self-Medication with Antibiotics for Protection against COVID-19: The Role of Psychological Distress, Knowledge of, and Experiences with Antibiotics. Antibiotics.

[33] Quincho-Lopez A., Benites-Ibarra C.A., Hilario-Gomez M.M., Quijano-Escate R., Taype-Rondan A. (2021). Self-medication practices to prevent or manage COVID-19: A systematic review. PLoS ONE.

[34] Karah N., Rafei R., Elamin W., Ghazy A., Abbara A., Hamze M., Uhlin B.E. (2020). Guideline for Urine Culture and Biochemical Identification of Bacterial Urinary Pathogens in Low-Resource Settings. Diagnostics.

[35] Igere B.E., Okoh A.I., Nwodo U.U. (2020). Antibiotic Susceptibility Testing (AST) Reports: A Basis for Environmental/Epidemiological Surveillance and Infection Control Amongst Environmental Vibrio cholerae. Int. J. Environ. Res. Public Health.

[36] Dhaliwal A. (2013). DNA extraction and purification. Mater. Methods.

